# Editor’s Note

**Published:** 2011-05

For manuscripts submitted after 31 May 2011, *EHP*’s page charges will increase to $35.00 per accepted manuscript page. In calculating page charges, we will include all pages of the final accepted Microsoft Word version of the manuscript (excluding the first three pages, which contain the title page, acknowledgments and support, and lists of keywords and abbreviations). We will continue to require authors to pay $500 for the first color figure (e.g., graphs, maps, schematics, photographs) and $100 for each additional color figure. [A figure includes all parts (e.g., A–D), but the completed figure must fit on one printed page or less.]

